# Infantile Nystagmus Syndrome—Associated Inherited Retinal Diseases: Perspectives from Gene Therapy Clinical Trials

**DOI:** 10.3390/life14111356

**Published:** 2024-10-23

**Authors:** Xiaoming Gong, Richard W. Hertle

**Affiliations:** 1 Department of Ophthalmology, Akron Children’s Hospital, Akron, OH 44308, USA; rhertle@akronchildrens.org; 2 Vision Center of Excellence, Rebecca D. Considine Research Institute, Akron Children’s Hospital, Akron, OH 44308, USA

**Keywords:** genotype–phenotype, inherited retinal disease, adeno-associated virus, gene therapy, clinical trials, infantile nystagmus syndrome, pediatric ophthalmology

## Abstract

Inherited retinal diseases (IRDs) are a clinically and genetically diverse group of progressive degenerative disorders that can result in severe visual impairment or complete blindness. Despite their predominantly monogenic inheritance patterns, the genetic complexity of over 300 identified disease-causing genes presents a significant challenge in correlating clinical phenotypes with genotypes. Achieving a molecular diagnosis is crucial for providing patients with definitive diagnostic clarity and facilitating access to emerging gene-based therapies and ongoing clinical trials. Recent advances in next-generation sequencing technologies have markedly enhanced our ability to identify genes and genetic defects leading to IRDs, thereby propelling the development of gene-based therapies. The clinical success of voretigene neparvovec (Luxturna), the first approved retinal gene therapy for *RPE65*-associated Leber congenital amaurosis (LCA), has spurred considerable research and development in gene-based therapies, highlighting the importance of reviewing the current status of gene therapy for IRDs, particularly those utilizing adeno-associated virus (AAV)-based therapies. As novel disease-causing mutations continue to be discovered and more targeted gene therapies are developed, integrating these treatment opportunities into the standard care for IRD patients becomes increasingly critical. This review provides an update on the diverse phenotypic–genotypic landscape of IRDs, with a specific focus on recent advances in the understanding of IRDs in children with infantile nystagmus syndrome (INS). We highlight the complexities of the genotypic–phenotypic landscape of INS-associated IRDs, including conditions such as achromatopsia, LCA, congenital stationary night blindness, and subtypes of retinitis pigmentosa. Additionally, we provide an updated overview of AAV-based gene therapies for these diseases and discuss the potential of gene-based therapies for underlying IRDs that lead to INS, offering a valuable resource for pediatric patients potentially eligible for ongoing clinical trials.

## 1. Introduction

Infantile nystagmus syndrome (INS) is a unique ocular motor disorder characterized by involuntary spontaneous oscillation of the eyes, typically associated with congenital or early onset defects in the visual sensory system within the first six months of life [1]. It is the most prevalent form of nystagmus in infancy and childhood, with an incidence of approximately 1.7 per 1000 live births [2,3]. INS is a common clinical sign of many ocular conditions in children and is frequently indicative of underlying ocular, neurological, and systemic diseases [4]. Evaluating INS in infants or children is challenging due to its association with both idiopathic oculomotor disorder and early-onset defects in the visual sensory system and systemic disorders [5]. Approximately 90% of INS cases are associated with visual sensory disorders that are either anatomical or functional [6], including various inherited retinal disorders (IRDs) [7].

**Figure 1:**
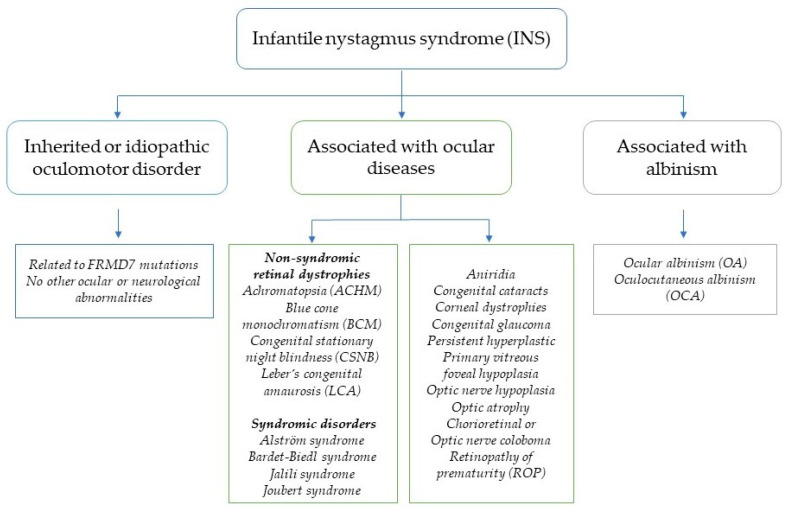
Clinical compositions of infantile nystagmus syndrome and its associated conditions.

INS can manifest as an isolated, inherited, or idiopathic oculomotor disorder without other associated ocular or neurological conditions. However, it is frequently observed alongside ocular disorders such as albinism, aniridia, congenital retinal dystrophies, early-onset retinal degeneration, congenital cataracts, optic nerve hypoplasia, and corneal dystrophies (Figure 1) [8,9]. INS may also be associated with neurological syndromes or conditions, including brain tumors, gliomas, spinocerebellar ataxia, and structural brain malformations [10]. Clinically, INS associated with IRDs is more common than idiopathic infantile nystagmus, although both share similar oculomotor characteristics. While the underlying “cause” of INS, such as a poorly calibrated smooth pursuit system, is consistent across cases, the diagnosis of associated visual and systemic conditions can be challenging due to the broad array of underlying genetic disorders. Given that approximately 70% of INS cases are associated with IRDs [11], identifying the genetic spectrum of IRDs in children with INS—referred to as INS-associated IRDs (INS-IRDs)—is crucial. This identification provides pediatric patients and their families with definitive diagnostic clarity, enables the early detection of INS-IRDs and tailored management strategies, and facilitates access to emerging gene-based therapies and the ever-increasing number of ongoing clinical trials.

IRDs are a clinically and genetically diverse group of conditions that lead to vision loss due to progressive retinal degeneration, often manifesting in childhood or early life and causing significant vision impairment and blindness in the pediatric population [12]. IRDs exhibit considerable genetic heterogeneity, with both monogenic and more complex inheritance patterns such as digenic, biallelic, and triallelic inheritance, particularly seen in conditions like achromatopsia, albinism, and Bardet–Biedl syndrome [13]. Over the past two decades, more than 100 causative genes have been identified in INS-associated IRDs [14], leading to significant phenotypic overlap (Tables S1 and S2). Mutations in the same gene can result in diverse phenotypes inherited through autosomal recessive, autosomal dominant, X-linked, or mitochondrial patterns, further complicating clinical diagnoses [15,16]. Despite this complexity, IRDs are excellent candidates for gene therapy, offering potential long-term solutions through localized delivery methods such as intravitreal, subretinal, or suprachoroidal injections [17]. The eye’s immune-privileged environment and the compartmentalized nature of ocular anatomy facilitate efficient gene delivery with minimal immune response [18,19]. Advances in adeno-associated virus (AAV)-based gene therapy have demonstrated safety and efficacy in treating various IRDs, as highlighted by the FDA approval of voretigene neparvovec-rzyl (Luxturna) for RPE65-associated retinal dystrophy [20]. With over 300 clinical trials underway, nearly 100 of which focus on ocular disorders, AAV-based gene therapies offer promising therapeutic approaches for monogenic and multigenic conditions. The following review will explore the genotype–phenotype complexities of INS-associated IRDs and provide an updated analysis of AAV-based gene therapies in pediatric ophthalmology, focusing on ongoing clinical trials targeting these conditions.

## 2. Phenotypic–Genotypic Complexity of IRDs in Children with INS

IRDs are primarily caused by mutations in more than 300 genes (RetNet; http://sph.uth.edu/retnet, accessed on 1 May 2024), each of which may harbor many disease-causing variants with distinct clinical phenotypes, underscoring the significant genetic diversity of these conditions. In children with INS, over 100 causal genes linked to IRDs have been identified, along with over 2500 mutations within these genes (RetinoGenetics: http://www.retinogenetics.org/, accessed on 1 June 2024). This extensive genetic variability makes INS-associated IRDs some of the most genetically heterogeneous Mendelian disorders, leading to a broad spectrum of clinical manifestations and symptoms. Examples of such IRDs include achromatopsia (ACHM), Leber congenital amaurosis (LCA), Bardet–Biedl syndrome (BBS), cone–rod dystrophies (CRD), and retinitis pigmentosa (RP). Clinically, INS-associated IRDs are classified into two phenotypic categories: non-syndromic and syndromic. Most cases are non-syndromic, primarily caused by mutations in retina-specific genes, and include conditions such as ACHM, LCA, RP, and congenital stationary night blindness (CSNB). Syndromic INS-associated IRDs, which involve multiple organ systems beyond the eyes, include disorders such as Alström syndrome, BBS, Jalili syndrome, and Joubert syndrome (Figure 1, Tables S1 and S2) [21].

The phenotypic presentation of INS-associated IRDs evolves over time, influenced by genetic and environmental factors, leading to significant variability even among family members with identical genetic mutations. The degree of rod and cone involvement drives clinical presentation, with predominant rod dysfunction causing impaired night vision and cone dysfunction leading to reduced visual acuity, color vision impairment, photophobia, and INS, especially in early-onset cases. In this review, we provide an in-depth analysis of the phenotypic–genotypic spectrum of common IRDs associated with INS, focusing on conditions such as LCA, ACHM, CSNB, various subtypes of RP, and X-linked retinoschisis.

### 2.1. The Phenotypic–Genotypic Spectrum of Leber Congenital Amaurosis (LCA)

LCA is one of the most severe forms of early-onset IRDs and the most prevalent type of INS-associated IRDs. It encompasses a group of severe, early-onset retinal disorders affecting the pediatric population [22]. Children with LCA frequently exhibit significant visual impairment from infancy or early childhood. This condition is characterized by almost non-detectable or severely abnormal full-field electroretinogram (ERG), infantile nystagmus (reflecting poor fixation ability), and amaurotic pupils (indicating poor pupillary light responses). While the retina may initially appear normal, pigmentary retinopathy reminiscent of retinitis pigmentosa is frequently observed later in childhood [23]. Other common phenotypic features include high refractive errors (hyperopia), sensitivity to light (photophobia), night blindness (nyctalopia), oculodigital reflex, peripheral chorioretinal atrophy, intraretinal pigment migration, drusen-like deposits, keratoconus, and cataracts [24]. From a clinical standpoint, LCA exhibits an extremely heterogeneous phenotype, ranging from an essentially normal retina to a variable degree of vessel attenuation, bone spicule pigmentation, pseudopapilledema, macular coloboma, salt and pepper pigmentation, yellow confluent peripheral spots, white retinal spots, preserved para-arteriolar retinal pigment epithelium (RPE), and Coats reaction, with some gene-specific features.

A milder form within this disease spectrum has been described using various terms, including early-onset severe retinal dystrophy (EOSRD), severe early-childhood-onset retinal dystrophy (SECORD), and early-onset retinitis pigmentosa (EORP) [25]. Prominent clinical features of severe-onset cone–rod dystrophy (CORD), which is related to early cone photoreceptor involvement, include reduced visual acuity and INS. The ocular fundus may appear normal or show signs like bull’s-eye or atrophic maculopathy, pigmented retinal stippling, or optic nerve pallor. EOSRD/SECORD is characterized by the onset of visual impairment typically emerging after infancy but before the age of five years, with variably preserved visual acuity and a better preserved full-field ERG response [25]. Collectively, LCA and EOSRD/SECORD are the most severe of the early onset forms of IRDs, affecting 20% of blind children and accounting for approximately 5% of all retinal dystrophies [24].

The molecular diagnosis of LCA/EOSRD is complicated by substantial genetic diversity and clinical variability. More than 28 genes have been implicated in these conditions, each associated with different phenotypes and pathogenic mechanisms that lead to various forms of retinal dysfunction [26]. These mechanisms impact several pathways, such as phototransduction, the visual cycle, and photoreceptor development and maintenance. The genes associated with LCA/EOSRD can be categorized based on the functions of their encoded proteins (Table 1). For example, *CCT2*, *CEP290*, *IQCB1*, *LCA5*, *RPGRIP1*, *SPATA7*, and *TULP1* are linked to intra-photoreceptor ciliary transport, whereas *CRB1*, *CRX*, *GDF6*, *CLUAP1*, and *PRPH2* are related to photoreceptor morphogenesis. *AIPL1*, *GUCY2D*, and *RD3* are involved in phototransduction, while *LRAT*, *RDH12*, and *RPE65* play roles in the visual cycle. *CABP4* and *KCNJ13* are important for signal transduction, *OTX2* for retinal development, *IMPDH1* for guanine synthesis, *MERTK* for outer segment phagocytosis, and *NMNAT1* for coenzyme NAD biosynthesis, whereas *DTHD1* has an unknown function. Most LCA/EOSRD genes follow an autosomal recessive inheritance pattern, though autosomal dominant mutations have been identified in *IMPDH1*, *OTX2*, and *CRX*. Certain genes, such as *GUCY2D*, *CEP290*, *NMNAT1,* and *AIPL1*, are predominantly associated with LCA, while others, including *RPE65* and *RDH12*, are more frequently linked to EOSRD [26].

The most prevalent causative genes associated with LCA/EOSRD include *GUCY2D*, *CEP290*, *CRB1*, *RDH12*, *NMNAT1,* and *RPE65*, which together account for 60% of cases. The variants in these genes lead to a broad spectrum of clinical phenotypes, which will be discussed in the following sections.

In LCA/EOSRD, *GUCY2D* mutations (associated with the LCA1 locus) are responsible for 6–21% of autosomal recessive LCA and up to 40% of autosomal dominant CORD and cone dystrophy [27]. The majority of *GUCY2D* mutations lead to a truncated protein, impairing guanylate cyclase 1 function. There are over 248 identified variants in the *GUCY2D* gene that cause the majority of autosomal recessive LCA/EOSRD, and 30 variants are linked to autosomal dominant CORD [28]. The majority of mutations in *GUCY2D* are missense, whereas nonsense, frameshift, and splice site mutations are also common. Patients with *GUCY2D*-related LCA often exhibit significantly reduced vision, INS, hyperopia, photophobia, and diminished or absent ERG responses. Despite severe visual impairment, these patients generally show normal fundi and a preserved central macular outer retinal structure on optical coherence tomography (OCT), unlike most other LCA/EOSRD genotypes [29].

Mutations in *CEP290* (associated with LCA10 locus) are responsible for >20% of LCA cases. These mutations are also implicated in a spectrum of more severe systemic conditions, including Joubert syndrome, Meckel–Gruber syndrome, Senior–Løken syndrome, and Bardet–Biedl syndrome [30]. Despite some variability among and within families, individuals with *CEP290* mutations typically exhibit a consistent and distinctive set of symptoms. Beyond the common LCA manifestations, these patients often experience significant early-onset vision loss, with most showing only light perception or complete blindness from birth. Specific LCA10 phenotypes include small atrophic spots in the RPE layer, a unique tapetal-like reflex, a prominent yellow scleral rim, pseudopapillary edema, and cyst-like macular lesions. Among the various *CEP290* variants, the pathogenic intronic nonsense mutation c.2991+1655A>G is the most prevalent, found in 60–80% of LCA patients with at least one affected allele [31]. This variant introduces a premature stop codon and a cryptic exon into the *CEP290* mRNA. However, as only about 50% of the mRNA includes the cryptic exon, this variant is considered a hypomorphic mutation, contributing to phenotypic variability [32].

Biallelic mutations in *CRB1* (associated with the LCA8 locus) have been associated with a wide range of retinal phenotypes, including early-onset severe macular atrophy, RP both with and without Coats-like exudative vasculopathy, CORD, and foveal retinoscisis. However, the most commonly reported is LCA or EOSRD [33]. Approximately 9–17% of LCA cases are related to *CRB1* mutations. Typical clinical and ophthalmological manifestations are progressive macular atrophy, with nummular pigmentation, and relative preservation of the para-arteriolar RPE. Unlike the retinal thinning typically seen in LCA, patients with *CRB1* mutations often exhibit increased retinal thickness with loss of the outer limiting membrane. Some patients may also develop retinal telangiectasis, exudative retinal detachment, and neovascular glaucoma [34].

Mutations in the *RDH12* gene (associated with the LCA13 locus) contribute to approximately 4–5% of all LCA cases and often lead to an EOSRD phenotype characterized by early-dense intraretinal pigmentation and maculopathy [35]. Patients with *RDH12* mutations typically have poor visual function early in life, show no ERG responses beyond 20 years of age, and do not experience photophobia. Clinical features include chorioretinopathy with dense pigmentation, bone spicules, minimal to no autofluorescence in the macula, and common night blindness. Spectral-domain OCT reveals significant macular thinning and a loss of foveal laminar structure. Hypomorphic alleles of *RDH12* are associated with milder retinopathy, primarily affecting the macula. The phenotypic range of *RDH12* mutations has recently been broadened to include later onset and milder presentations [36]. There are currently more than 130 disease-causing mutations in *RDH12* that have been identified in association with LCA, RP, and other retinal dystrophy phenotypes. The mutation spectrum includes missense mutations, truncating mutations (nonsense, frameshift, and consensus splice sites), and gross deletions.

Mutations in *NMNAT1* (associated with the LCA9 Locus) account for 4–14% of LCA cases. There are 75 identified mutations in *NMNAT1*, including 54 missense mutations, seven frameshift indel, six nonsense mutation, three intronic mutations, and five other mutations, such as exon deletion, exon duplication, and large duplication [37]. Extreme early onset and rapid progression are characteristics of *NMNAT1*-associated LCA. Patients with *NMNAT1* mutations show severe visual impairment at birth and coloboma-like macular dystrophy with peripheral retinal degeneration and optic atrophy since early infancy. Attenuated vessels, pallor optic disc, and peripheral pigmentation are observed during fundus examination [38]. No correlation has been found between genotype and clinical phenotype.

LCA associated with *RPE65* (the LCA2 locus) is particularly notable due to available treatments for patients with biallelic variants in this gene. Pathogenic mutations in the *RPE65* gene account for 8–16% of LCA cases and around 2% of recessive RP cases [39]. Most children with *RPE65*-associated retinopathy present with severe night blindness from birth. Patients who carried at least one nonsense variant had more progressive deterioration in retinal sensitivity. The clinical presentation aligns with that typical of LCA features, as mentioned above. Additionally, patients with *RPE65*-related retinopathy may also present with SECORD/EOSRD.

### 2.2. Achromatopsia (ACHM)

Achromatopsia (ACHM) is a congenital recessive IRD that primarily affects the cone cells in the retina [40]. This disorder is defined by dysfunctional cone photoreceptors and can manifest in either complete or incomplete forms. In complete ACHM, patients experience deficits in all three color perception axes (red, green, and blue) and typically exhibit significantly reduced visual acuity, infantile nystagmus, sensitivity to light (photophobia), and a congenital inability to differentiate colors. In the incomplete form, some cone function persists, resulting in better visual acuity and partial color discernment. Both forms of ACHM are marked by difficulties in color differentiation across all three color vision axes, INS with a dual jerk waveform, photophobia, central visual field defects (scotomata), and frequently hypermetropic refractive errors [41].

The majority of individuals with ACHM has the complete form. Full-field ERG usually shows no detectable cone-mediated responses, but rod responses remain normal or near-normal [42]. OCT can present a broad spectrum of findings, from a normal ellipsoid zone (EZ) to complete outer retinal atrophy, including the retinal pigment epithelium (RPE). OCT may also reveal hypo-reflective cavities in place of the EZ [43]. Fundus autofluorescence (FAF) imaging can show hyper-autofluorescence in zones with preserved EZ, indicating early disease stages before photoreceptor loss, or hypo-autofluorescence in areas with photoreceptor loss or RPE atrophy [44].

ACHM is a genetically well characterized condition. Six genes are currently recognized as causes of ACHM. Five of these genes, including cyclic nucleotide-gated cation channel alpha-3 (*CNGA3*), cyclic nucleotide-gated cation channel beta-3 (*CNGB3*), guanine nucleotide-binding protein G (t) subunit alpha-2 (*GNAT2*), the catalytic alpha-subunit of cone cyclic nucleotide phosphodiesterase (*PDE6C*), and the inhibitory gamma-subunit of cone phosphodiesterase (*PDE6H*), encode critical elements of the cone phototransduction pathway, which converts light signals into electrical signals. The sixth gene, activating transcription factor 6 (*ATF6*), is a more recently identified gene that codes for a transcription factor crucial for cone photoreceptor development [45]. No clear genotype–phenotype correlation has been observed in ACHM.

Genetic studies of ACHM patients have shown that many cases have pathogenic mutations in the *CNGA3* and *CNGB3* genes. Loss-of-function mutations in *CNGA3* and *CNGB3* account for over 70% of ACHM cases [46]. More than 150 mutations in *CNGA3* and 140 mutations in *CNGB3* have been linked to ACHM [47]. The majority of CNGB3 mutations are nonsense, frameshift, or splicing variants resulting in truncated or dysfunctional CNG channel proteins, while most *CNGA3* mutations are missense variants that impair or abolish CNG channel function. Patients with homozygous or compound heterozygous mutations exhibit typical ACHM symptoms, whereas carriers maintain normal vision. Interestingly, some ACHM patients exhibit a digenic or triallelic inheritance pattern involving mutations in both genes [48].

Mutations in *GNAT2* are responsible for a rare form (<2%) of ACHM [49]. Similarly rare are forms caused by mutations in *PDE6C* and *PDE6H* [50]. *ATF6* encodes a transmembrane transcription factor that activates target genes involved in the unfolded protein response during endoplasmic reticulum (ER) stress. This function potentially increases susceptibility to ER-stress-induced damage and death during cone photoreceptor development. The majority of complete ACHM patients with pathogenic mutations in *ATF6*, *CNGA3*, *CNGB3*, *GNAT2*, and *PDE6C* exhibit similar clinical phenotypes. Although a significant genotype–phenotype correlation cannot be established, the frequent observation of foveal hypoplasia with a poorly formed or absent foveal pit in *ATF6*-associated ACHM suggests the critical role of this gene in foveal development [45]. Pathogenic mutations in the *ATF6* gene predominantly include truncating mutations such as splice site, nonsense, and frameshift mutations, whereas missense mutations have also been reported.

### 2.3. Congenital Stationary Night Blindness (CSNB)

Congenital stationary night blindness (CSNB) is a group of IRDs that primarily manifest as night blindness in childhood. CSNB displays diverse genetic, electrophysical, and clinical characteristics. This retinal dystrophy is defined by the dysfunction of rod photoreceptors and impaired signal transduction between photoreceptor cells and bipolar cells. Clinically, patients present with night blindness, myopia, strabismus, and/or nystagmus. The nystagmus associated with CSNB is typically described as pendular, dysconjugate, and oblique, characterized by high frequency and low amplitude movements [51]. Several of these symptoms overlap with other IRDs, such as cone–rod dystrophies. CSNB can be classified into four types: Riggs, Schubert–Bornschein, fundus albipunctatus, and Oguchi disease [52]. On fundus examination, fundus albipunctatus is identified by the presence of small white dots scattered across the posterior pole, sparing the fovea. Oguchi disease, in contrast, features a distinctive gray–white metallic sheen on the retina, which disappears after dark adaptation—a phenomenon known as the Mizuo–Nakamura phenomenon. Unlike these two diseases, the Riggs and Schubert–Borstein types have normal fundi and can be distinguished using full-field ERG. In Riggs-type CSNB, a non-recordable rod ERG and a reduced a-wave in the dark-adapted combined rod–cone response indicates impaired rod phototransduction. The Schubert–Bornschein type of CSNB, on the other hand, is characterized by a normal dark-adapted a-wave but a severely reduced b-wave, resulting in an ”electronegative” ERG. The Schubert–Bornschein type can be further subdivided into complete and incomplete forms. Complete CSNB presents with a normal a-wave and a reduced or absent b-wave under scotopic conditions, while photopic conditions elicit a near-normal b-wave in response to bright flashes or 30 Hz flicker stimuli. In contrast, incomplete CSNB shows a reduced b-wave under scotopic conditions but also has a near-normal response to bright flashes or flickers at 30 Hz under photopic conditions.

CSNB follows an autosomal dominant inheritance pattern in 2% of cases, an autosomal recessive pattern in 40%, and an X-linked recessive pattern in 58% [53]. To date, more than 300 mutations in 18 genes have been associated with CSNB. Specifically, GNAT1, PDE6B, and RHO are implicated in the autosomal dominant form, while CACNA1F and NYX are linked to the X-linked form. The autosomal recessive form involves 13 genes, including CABP4, GNAT1, GNB3, GPR179, GRK1, GRM6, LRIT3, RDH5, RIMS2, RPE65, SAG, SLC24A1, and TRPM1 (Table 2). The complete form of CSNB is associated with mutations in NYX, GRM6, TRPM1, SLCC24A1, GPR179, LRIT3, GNAT1, GNB3, RHO, and RPE65, which all lead to a loss of function of rod and cone ON bipolar cells. This results in a characteristic squared-off appearance of the b-wave, with a normal OFF response driven by cone OFF bipolar cells. Incomplete CSNB, attributed to mutations in CACNA1F, CABP4, and PDE6B, presents with reduced a-wave and b-wave amplitudes in light-adapted ERG and markedly decreased 30 Hz flicker amplitude, indicating the severe dysfunction of both rods and cones.

### 2.4. Retinitis Pigmentosa (RP)

RP is a heterogeneous group of IRDs caused by the progressive degeneration of rod and cone photoreceptors. It is the most frequent form of IRD, affecting between one in 3000 to 4000 individuals. Clinically, RP is characterized by night blindness, peripheral vision loss, and ultimately, total blindness [54]. The disease often begins with the degeneration of rod photoreceptors, which predominantly affects night vision and adaptation to low light. Early symptoms include night blindness (nyctalopia) and difficulties adjusting to changes in light sensitivity. As rod degeneration advances, it causes a progressive constriction of the visual field, initially affecting mid-peripheral vision and eventually leading to “tunnel vision” as the central vision becomes compromised [55]. In later stages, RP may present as cone–rod degeneration and cone dystrophy, where cone photoreceptors are primarily affected, resulting in significant loss of visual acuity. Some RP subtypes exhibit concurrent degeneration of both rod and cone photoreceptors.

The underlying molecular causes of RP are highly intricate, involving mutations in over 200 genes, which account for its genetic diversity and clinical variability. RP can be categorized into three main inheritance patterns [56]: autosomal dominant RP (adRP), accounting for 15–25% of cases; autosomal recessive RP (arRP), comprising 5–20% of cases; and X-linked RP (XL-RP), representing 10–15% of RP cases. Approximately 40–50% of RP cases lack a clear inheritance pattern and are thus classified as sporadic.

Several key genes are frequently mutated in RP, including *RHO* (rhodopsin), responsible for about 25% of adRP cases; *USH2A*, associated with 20% of arRP cases; and *RPGR* (retinitis pigmentosa GTPase regulator), which accounts for over 80% of XL-RP cases. XL-RP is one of the most severe forms of rod–cone dystrophy, accounting for 10–20% of all RP cases [57]. So far, mutations in three genes have been identified as causes of XL-RP: *RPGR*, retinitis pigmentosa 2(*RP2*), and oral–facial–digital syndrome type 1 (*OFD1*).

Mutations in *RPGR*, located in the RP3 region of Xp21.1, is the most common genetic cause of XL-RP. In addition to typical RP symptoms, patients with *RPGR* mutations often present with severe myopia and can display sectoral RP [58]. The severity in female carriers varies widely, ranging from no symptoms to severe RP, with some displaying a tapetal-like reflex (TLR). Microperimetry has shown that most RPGR patients experience a rapid decline in retinal sensitivity during their second and third decades of life [59].

Mutations in *RP2* are the second most common genetic cause of XL-RP, accounting for 5–20% of cases. The majority of affected males typically show early-onset, severe retinal degeneration, with significant macular involvement and a complete loss of the foveal photoreceptor layer by the third decade of life [60]. Female carriers of *RP2* mutations exhibit a range of symptoms, from a normal fundus to TLR to peripheral pigmentary changes, generally with a favorable prognosis.

The *RPGR* gene comprises nineteen exons and undergoes extensive alternative splicing, resulting in two main isoforms: the full-length RPGR^1−19^ isoform and the RPGR^ORF1*5*^ isoform. The full-length RPGR^1−19^ isoform, which contains all 19 exons, is widely expressed in various tissues. Conversely, the RPGR^ORF15^ isoform consists of exons 1 to 14 from RPGR^1−19^ plus a unique 3′ terminal exon (ORF15, open reading frame 15) that encodes two critical domains: a regulator of the chromosome condensation 1 (RCC1)-like domain (rich in glycine and glutamic acid) and a C-terminal basic domain with homology to tubulins [61]. The RPGR^ORF15^ isoform is predominantly expressed in rod photoreceptor outer segments. Truncations in this terminal exon disrupt the vital C-terminal domain, with about 80% of *RPGR* frameshift mutations occurring in the ORF15 region [62]. The most frequent pathogenic mutations in RPGR^ORF1*5*^ are small deletions, whereas splice site mutations are rare across the *RPGR* gene [63]. Pathogenic mutations in exons 1–14 of *RPGR* can affect the RCC1-like domain, which is crucial for protein stability and interactions, leading to a loss of function and progressive retinal degeneration [64]. Mutations in the exons specific to the constitutive variant are primarily associated with XL-RP, while mutations in the ORF15 exon, a known mutational hotspot, are also associated with cone dystrophy (COD) and cone–rod dystrophy (CRD) [65].

Mutations in the *RPGR* gene lead to various phenotypes, such as rod–cone dystrophy (RCD) (70%), CRD (6–23%), and COD (7%) [66]. Most mutations in exons 1–14 and the 5′end of ORF15 are associated with RCD, whereas those causing COD or CRD tend to be located at the 3′end of the ORF15 [67]. However, there is no clear consensus on genotype–phenotype correlation. Some studies indicate that mutations in exons 1–14 are associated with more severe disease phenotypes than those in ORF15 [68], while others suggest the opposite [69].

### 2.5. X-Linked Retinoschisis (XL-RS)

X-linked retinoschisis (XL-RS) is an IRD caused by mutations in the *retinoschisin 1* (RS1) gene, which encodes Retinoschisin-1, a protein essential for the retinal structure and cell adhesion [70]. It is the most prevalent form of juvenile macular degeneration in males [71]. Symptoms generally manifest in early childhood and include reduced visual acuity, strabismus, anisometropia, and progressive vision deterioration. While most patients are diagnosed within the first decade of life, severe visual loss in infancy may present with INS and poor fixation [72,73]. The characteristic clinical sign of XL-RS is foveal schisis, which appears as small cystoid spaces in a spoke-wheel configuration on fundoscopy. Approximately half of the affected males also exhibit peripheral retinoschisis, pigmentary changes, white spiculations, and a metallic sheen. This disorder disrupts the communication between photoreceptors and bipolar cells, causing a diminished b-wave and often an electro-negative ERG [74].

XL-RS typically leads to declining visual acuity in the first or second decade of life, with progressive macular atrophy continuing until the fifth or sixth decade, potentially resulting in legal blindness [75]. ERG is a critical diagnostic tool, showing a reduced b-wave with a relatively preserved a-wave, characteristic of an “electronegative” ERG. Multimodal imaging is often necessary to identify macular schisis, with structural OCT revealing schisis cavities and OCT angiography showing foveal vascular impairment.

## 3. AAV-Based Gene Therapy for INS-Associated IRDs

Advances in next-generation sequencing (NGS) have significantly enhanced our understanding of the genetic underpinnings of IRDs in children with INS. These developments facilitate the early diagnosis of INS-associated IRDs and allow for the timely recruitment of eligible patients for gene-based therapy clinical trials during the early stages of the disease. Gene therapy, an innovative treatment modality, involves modifying or replacing defective genes or delivering therapeutic molecules such as small interfering RNAs (siRNAs) and proteins [76]. Various strategies are under investigation for delivering therapeutic genes to target cells in vivo, including both viral and non-viral vectors. Among these, viral vectors, particularly adeno-associated viruses (AAVs), are the most commonly employed in both preclinical studies and clinical applications [77].

Discovered in the 1960s, AAVs have emerged as ideal vectors for therapeutic gene delivery due to their minimal integration into host genomes, ability to transduce non-dividing cells, prolonged expression, low immunogenicity, versatile tissue tropism, and relatively ease of production [78,79]. AAVs exhibit a favorable benefit–risk profile, providing long-term gene expression tailored to specific serotypes and tissue tropism [80]. Recombinant AAV vectors (rAAVs), including serotypes AAV2, 5, 6, 8, and 9, along with their engineered capsid variants such as AAV2tYF, AAV2.7m8, and 4D-R100, particularly show promise for in vivo retinal gene therapy, especially for treating various IRDs [20,81,82]. The safety and efficacy of AAV-based retinal gene therapies have been well documented in numerous clinical studies, including the prominent example of Luxturna [20]. Despite their many advantages, AAV vectors have a limited packaging capacity of approximately 4.7 kilobases, posing a challenge for large gene delivery [83]. To overcome this constraint, the co-delivery of dual or triple AAVs, such as those explored for delivering the *ABCA4* gene in Stargardt disease, has been developed [84,85]. However, these delivery systems face significant challenges, particularly the requirement for the co-transduction of target cells.

Depending on the genotypic and phenotypic spectrum of INS-IRDs, as well as the pathogenic mechanisms involved, various DNA- or RNA-based therapeutic strategies are employed in AAV-mediated gene therapy. The therapeutic approach is generally tailored to the specific mutation. For loss-of-function mutations—commonly found in autosomal recessive or X-linked recessive IRDs—AAV-based gene augmentation or replacement is used to deliver a functional copy of the affected gene to the retina. This strategy’s efficacy has been validated, notably with the FDA’s approval of Luxturna for RPE65-related retinal dystrophy, and is supported by multiple clinical trials. The developmental pathway established by Luxturna has been adapted to explore additional gene therapy techniques for IRDs, such as mitochondrial gene delivery, gene editing tools, RNA interference (RNAi), and microRNA therapies.

In contrast, some autosomal dominant IRDs involve gain-of-function mutations or dominant-negative mutations, which lead to toxic effects. The therapeutic goal for these cases is to inhibit the expression of the mutated genes. Ribozyme-based, interfering RNA-based, and CRISPR-based methods have been developed for this purpose. These approaches can target the disease-causing gene through two strategies: allele-specific inhibition, which selectively silences the mutant allele while allowing the normal allele to be expressed; and allele non-specific inhibition combined with gene augmentation, where both alleles are silenced, and a normal gene is introduced using dual AAV vectors. While this second method is broadly applicable, it faces challenges with vector packaging constraints [86,87]. Additionally, given the complexity and cost of developing therapies for each disease-causing gene, gene-agnostic approaches like optogenetics and gene modifiers offer a promising alternative by targeting common pathways across multiple IRDs, potentially reducing development costs and broadening treatment access [88,89].

### 3.1. AAV-Based Gene Augmentation Therapy for INS-Associated IRDs in Clinical Trials

As of the second quarter of 2024, more than 60 clinical trials investigating gene therapy for IRDs are listed on Clinicaltrials.gov, accessed on 1 July 2024. This includes 29 trials for RP, 10 for LCA, 4 for achromatopsia, and 6 for X-linked retinoschisis (Table 3). Many of these trials utilize AAV-based gene augmentation or replacement approaches. Additionally, RNA-based strategies, such as RNA interference (RNAi) or microRNA, antisense oligonucleotides (AON), and gene editing techniques like CRISPR-based therapy, are explored for the treatment of IRDs. This review focuses on recent and ongoing AAV-based gene therapy clinical trials targeting INS-associated IRDs. Additionally, we briefly discuss the current status of RNA-based and CRISPR-based therapeutic strategies for IRDs.

#### 3.1.1. AAV-Based Gene Augmentation Therapy for Achromatopsia

Achromatopsia (ACHM) is a recessive disorder resulting from loss-of-function mutations in any of the six genes responsible for cone photoreceptor function. Currently, AAV-based gene therapies are being developed focusing on two of the most frequently affected genes, *CNGA3* and *CNGB3* [90]. The initial clinical trial assessed the safety and efficacy of the subretinal injection of AAV8.hCNGA3 in nine individuals with CNGA3-associated ACHM (NCT02610582) [91]. Over a one-year period, the treatment was well tolerated, with no serious adverse events reported. Despite the congenital loss of cone-driven light signaling in patients with CNGA3-ACHM, AAV8.CNGA3 treatment led to improvements in secondary endpoints related to cone function. These include increases in visual acuity and contrast sensitivity compared to the baseline in all nine treated patients, persisting for at least three years post-treatment [91]. An ongoing phase IIb clinical trial is targeting the treatment of the second eye in the initial patients and the treatment of children aged 6–12 years.

At present, four additional phase I/II AAV-based gene therapy trials are actively recruiting adults and children with CNGA3- and CNGB3-related ACHM. Two phase I/II open-label, dose-escalation trials for CNGA3 (NCT02935517) and CNGB3 (NCT02599922) are utilizing subretinal injections of AAV2 variant vectors. These vectors employ an engineered cone opsin promoter to drive the expression of CNGA3 (rAAV2tYF-PR1.7-hCNGA3) in patients with CNGA3-associated ACHM and CNGB3 (rAAV2tYF-PR1.7-hCNGB3) in patients with CNGB3-associated ACHM. Participants in both studies were sequentially assigned to one of four dose groups. The current data suggest that rAAV2tYF-PR1.7-hCNGB3 treatment has improved photosensitivity in some patients, while the effect of rAAV2tYF-PR1.7-hCNGA3 appears less encouraging.

Additionally, two similar phase I/II, open-label, dose-escalation clinical trials are underway for CNGA3 (NCT03758404) and CNGB3 (NCT03001310). One trial is evaluating AAV2/8-hG1.7p.coCNGA3 (AAV-CNGA3) in adults and children with CNGA3-associated ACHM, while the other is assessing AAV2/8-hG1.7p.coCNGB3 (AAV-CNGB3) in adults and children with CNGB3-associated ACHM. The primary outcome measure for each of these trials is the incidence of treatment-related adverse events at six months. Secondary outcomes include improvements in visual function, retinal function, and quality of life.

#### 3.1.2. AAV-Based Gene Augmentation Therapy for X-Linked Retinoschisis

There are currently two ongoing clinical trials using AAV-based gene augmentation therapy for X-linked RS. Due to the retinal fragility and tendency for retinal detachments associated with XL-RS, the AAV vectors are administered via intravitreal (IVT) injection rather than subretinal injection, which is the method typically used in other retinal gene therapy. Preclinical research has demonstrated that the internal limiting membrane (ILM), which poses a key barrier to IVT AAV-mediated gene transfer, is compromised in XL-RS, thereby enhancing the effectiveness of IVT injections [92].

The National Eye Institute (NEI) conducted a phase I/IIa trial (NCT02317887) to assess the safety and efficacy of AAV8-RS1 gene therapy in nine male patients with XLRS. At the 18-month follow-up, the treatment and vector were generally well tolerated. However, functional outcome measures such as BCVA, microperimetry retinal sensitivity, and ERG response showed no significant visual improvements from baseline. One patient who receiving a high dose (1 × 10^11^ vg compared to 1 × 10^10^ vg) exhibited temporary closure of macular cavities two weeks post-injection, likely due to RS1 protein activity [92].

Another phase I/II trial (NCT02416622) utilized an AAV2 vector variant encoding the RS-1 gene (rAAV2tYF-CB-hRS1) for IVT delivery. Preclinical data suggested AAV2 more effectively transduces ganglion cells than AAV8 following IVT injection [93]. The six-month results indicated a favorable safety profile but no functional improvements, and no measurable benefits were observed at the 12-month mark [94]. Given the slow progression of XL-RS, longer-term follow-up is essential to determine potential functional gains. Recently, a new phase I/II open-label trial (NCT05878860) has commenced. This trial aims to evaluate the safety and tolerability of ATSN-201 (AAV.SPR-hRS1) in male patients aged 6 to 64 with XL-RS.

#### 3.1.3. AAV-Based Gene Augmentation Therapy for Leber Congenital Amaurosis

LCA2 resulting from RPE65-mutation was the first IRD to undergo gene therapy trials. Biallelic disease-causing variants in RPE65 are responsible for 5–10% of LCA cases. To date, more than 10 clinical trials have evaluated the efficacy and safety of a single subretinal injection of AAV2-hRPE65 in patients with confirmed biallelic RPE65 mutations. These studies primarily assess functional vision improvements rather than structural changes.

A phase I/II trial (NCT00481546) involving 15 young patients receiving subretinal rAAV2-CBSB-hRPE65 injection reported no serious adverse events, with all patients experiencing varying degrees of visual function improvement, which persisted for up to 36 months post-injection [95]. In a phase III randomized controlled trial (RCT) (NCT00999609), 31 participants (21 in the intervention group and 10 in the control group) with confirmed RPE65-associated retinal dystrophy received bilateral sequential subretinal injections of AAV2-hRPE65v2 (voretigene neparvovec, Luxturna) [96,97]. This phase III trial demonstrated significant improvements in multi-luminance mobility test (MLMT) scores, the full-field light sensitivity threshold (FST), and visual fields (VFs) in the treatment group compared to the controls. These clinically meaningful effects were maintained for at least one year, with no serious adverse events or harmful immune responses reported [96].

A recent review of six studies (five prospective and one RCT) on RPE65-LCA gene therapy indicated that visual function improvements were generally limited to two years post-treatment [98]. However, BCVA and FST were the only visual function outcomes consistently analyzed across these studies. Other visual function measures, such as MLMT and VF testing, might show sustained improvement beyond two years. While previous safety analyses showed a favorable adverse event profile, they noted a thinning of central retinal thickness in treated eyes compared to untreated eyes 2–3 years after the intervention [98,99].

Beyond *RPE65*, several AAV-based gene therapy trials are in progress for LCA, targeting different gene mutations including *GUCY2D*, *CRB1*, and *CEP290*. *GUCY2D* is associated with LCA1, with biallelic mutations accounting for 10% to 20% of cases. A phase I/II trial (NCT03920007) was launched to evaluate the safety and tolerability of ascending doses of AAV8-hGRK1-GUCY2D, administered via subretinal injection in patients with GUCY2D-associated LCA1 [100]. This ongoing trial enrolled 15 patients aged six years or older, with nine in dose-escalation groups and six in dose-expansion groups. Early results from the first three patients treated with AAV8-hGRK1-GUCY2D indicated rod photoreceptor vision improvement by FST testing in the treated eye, with one patient experiencing a 0.3 LogMAR improvement in vision [100]. Two patients showed improvements in visual function and functional vision, reaching more than 3 log units and nearing healthy rod vision [101].

#### 3.1.4. AAV-Based Gene Augmentation Therapy for X-Linked Retinitis Pigmentosa

XL-RP is primarily driven by mutations in several genes, with RPGR accounting for 80% of all XL-RP cases and approximately 11% of all RP cases [62]. The majority of XL-RP patients have a mutated RPGR gene, making it a key target for AAV-based gene therapy. The initial phase I/II clinical trial for XL-RP aimed to evaluate the safety and efficacy of a codon-optimized AAV8-RPGR, known as cotoretigene tiparvovec (BIIB112) (NCT03116113, XIRIUS). This trial involved subretinal injections of low, intermediate, and high doses of BIIB112 in 18 XL-RP patients. The six-month follow-up indicated no significant dose-limiting safety issues, except for steroid-responsive retinal inflammation at higher doses [102]. Some patients showed improved visual acuity and retinal structure stability. A post hoc analysis of the XIRIUS and natural history study (XOLARIS) for XL-RP revealed that the four patients who received the highest doses of BIIB112 exhibited early improvements in retinal sensitivity and low-luminance visual acuity at one year [102]. However, in the following phase II/III of the XIRIUS study, the primary endpoint of statistically significant improvement in microperimetry was not achieved. Positive trends were observed in the visual acuity of treated eyes under low luminance vision measurements. Patients exiting the trial have been offered enrollment in a separate phase III trial (NCT03584165) to monitor long-term outcomes over five years.

Additionally, two other phase I/II clinical trials have been conducted to evaluate AAV-based gene augmentation therapy for XL-RP [103]. One phase I/II, open-label, dose escalation study (NCT03252847) assessed the efficacy and safety of AAV5-hRKp.RPGR over 18 months in adults and children over 5 years old with XL-RP. The one-year data from this trial demonstrated that AAV5-hRKp.RPGR was generally well tolerated and led to significant vision improvements. Currently, a phase III randomized, controlled study (NCT04671433) of AAV5-hRKp.RPGR and a phase III trial assessing patient safety for up to 60 months (NCT04312672) are underway.

The second trial, which is also an open-label phase I/II dose escalation study (NCT03316560), involves 29 patients receiving subretinal injections of rAAV2tYF-GRK1-RPGR. This gene therapy, driven by the GRK1 promoter for targeted photoreceptor expression, is being assessed for safety and efficacy in both adults and children with XL-RP. Participants were divided into five cohorts: 21 received injections in the central macula, while 8 were injected in the peripheral retinal regions. Surgery-related adverse events were mild to moderate. The twelve-month follow-up data in male patients with XL-RP indicated significant improvements in visual function, particularly retinal sensitivity. An ongoing phase II/III trial (NCT04850118) aims to further evaluate the therapy’s efficacy, safety, and tolerability by comparing two doses of rAAV2tYF-GRK1-RPGR with an untreated control group. Additionally, a phase III trial (NCT04794101) involving the bilateral administration of two vector genome doses (2 × 10^11^ and 4 × 10^11^) is currently in progress, along with a follow-up study.

While other RPGR clinical trials utilize subretinal injections for AAV-mediated RPGR delivery, 4DMT employs intravitreal injections. The EXCEL trial (NCT04517149), a phase I/II dose-escalation study, used a capsid-engineered AAV2 vector (4D-R100) developed by 4DMT to deliver a functional copy of the RPGR gene to the retina. The study aimed to evaluate the efficacy, safety, and maximum tolerated dose of 4D-125 in XL-RP patients. In the phase I portion, participants received one of two doses (3 × 10^11^ vg/eye or 1 × 10^12^ vg/eye), and in the phase II expansion, the higher dose (1 × 10^12^ vg/eye) was administered. Results from the phase I/II trial in advanced RPGR patients, with limited or no measurable photoreceptor regions and low or no retinal sensitivity, showed no dose-limiting toxicity or serious adverse events. Two patients exhibited increased retinal sensitivity in treated eyes at 6 and 9 months follow-up.

#### 3.1.5. AAV-Based Gene Therapy for MERTK-, RLBP1-, and PDE6B-Associated RP

MERTK (MER Proto-Oncogene, Tyrosine Kinase) is associated with RP and encodes a transmembrane protein found in RPE cells. This protein plays a role in the uptake of photoreceptor outer segments by RPE cells. Mutations in the *MERTK* gene hinder this phagocytic process, leading to photoreceptor cell degeneration and ultimately RP [104]. A phase I clinical trial (NCT01482195) evaluated the effects of subretinal injections of rAAV2-VMD2-hMERTK in six patients with MERTK-associated RP [105]. Over a two-year follow-up, no significant ocular or systemic adverse events were observed. Three patients exhibited measurable visual acuity improvements, but only one patient maintained these gains over two years. Two patients who had temporary visual improvements developed bilateral cataracts, which may have influenced the assessment of the treatment’s efficacy.

Retinaldehyde binding protein 1 (RLBP1) is a gene associated with autosomal recessive RP, encoding a protein expressed by Müller glial and RPE cells [106]. Mutations in *RLBP1* impair the visual cycle between the RPE and photoreceptors, similar to the dysfunction seen in RPE65-associated LCA. An ongoing phase I/II clinical trial (NCT03374657) is examining the safety, tolerability, and efficacy of subretinal administration of AAV8-RLBP1 (CPK850) in patients with RLBP1-RP, though no data are yet available.

Phosphodiesterase 6 (PDE6) is a complex enzyme that hydrolyzes cGMP in rod photoreceptors, reducing its concentration in response to light activation of the G-protein-coupled receptor during phototransduction. The PDE6 complex consists of alpha, beta, and two gamma subunits, with mutations in the alpha and beta subunits each accounting for approximately 4% of all RP cases [107]. A phase I/II clinical trial (NCT03328130) evaluated the safety and efficacy of AAV5-hPDE6B via subretinal injections at three different doses. The trial showed significant visual function improvement at the highest dose with a favorable safety profile [108]. Recently, STZ eyetrial has been conducting a phase I/II study with their vector, rAAV-hPDE6A, to evaluate its safety and efficacy in patients with *PDE6A* mutations (NCT04611503, PIGMENT). No data are currently available for this trial.

### 3.2. AAV-Based Gene Silencing for INS-Associated IRDs

As previously mentioned, gene augmentation using AAV vectors is a well-established method for treating AR-associated IRDs. However, this technique is not effective for AD-associated retinal diseases, which constitute approximately 15–20% of all IRDs. In such cases, delivering a normal copy of the mutated gene does not suffice; instead, it is necessary to silence the dominant allele. Various methods can achieve this silencing. One option is RNA-targeting therapy, including RNA interference (RNAi), microRNAs, and antisense oligonucleotides (AONs), which work to silence gene expression. For IRDs caused by splicing defects, AONs have been effective in restoring proper pre-mRNA splicing, such as in the case of the common *CEP290* gene mutation (c.2991+1655A>G) associated with LCA10 [109,110]. In a phase I/II clinical trial (NCT03140969), an AON-based drug (QR-110) was administered via intravitreal injections to 10 patients with CEP290-LCA every three months for up to 12 months [109]. Results from the phase I/II trial demonstrated significant improvements across all outcome measures, including BCVA, OCT structure, mobility, and nystagmus measurements [111]. Following a post-surgical follow-up of at least three months, the study is now enrolling patients with the compound heterozygous or homozygous intron 26 variant c.2991+1655A>G in *CEP290* for a phase III trial (NCT03913143). Additionally, AON therapy has shown potential in correcting splicing signals to induce in-frame skipping of exon 13 in the *USH2A* gene, resulting in the production of a shorter yet functional protein in Usher syndrome [112].

Gene editing techniques, such as CRISPR/Cas9 and transcription activator-like effector nucleases (TALENs), can also be utilized to silence mutant alleles that cause toxic gain-of-function effects. The CRISPR/Cas9 method has been successfully applied in studies on autosomal dominant RP associated with rhodopsin (*RHO*) mutations (RHO-adRP) [113,114]. Moreover, correcting splice defects using this strategy has been effective in preclinical models with a deep intronic mutation in *CEP290*, showing both safety and efficacy in the retina [115]. The development of a self-limiting CRISPR/Cas9 system has further minimized the immune response by reducing the duration of Cas9 expression [115].

### 3.3. AAV-Based Gene Editing for INS-Associated IRDs

Precise genome editing technologies, particularly the CRISPR/Cas9 system, are becoming promising alternatives to traditional gene therapy approaches. The CRISPR/Cas9 system is especially valuable for therapeutic editing due to its adaptability in targeting virtually any gene. This system includes CRISPR RNA, which contains spacer sequences that pair with the target DNA sequence, and a tracer-RNA that binds to Cas endonucleases. Accurate target recognition relies on the complementary base pairing between the guide RNA (gRNA) and the target genomic region, necessitating the presence of a protospacer adjacent motif (PAM). PAM sequences are essential for Cas proteins to locate their target DNA, and different Cas variants have been designed to recognize a variety of PAM sequences, increasing the system’s flexibility [116,117].

Cas endonuclease, such as *Streptococcus pyogenes* (SpCas9) or *Staphylococcus aureus* (SaCas9), are guided to specific DNA target sites in the genome through a gRNA, inducing DNA double-strand breaks (DSBs). These DSBs subsequently activate one of several DNA repair mechanisms: non-homologous end joining (NHEJ), homology-directed repair (HDR), or microhomology-mediated end joining (MMEJ) [118]. Among these, the NHEJ pathway is the most readily used by cells to repair DSBs. During the repair process, NHEJ often introduces random elements into the genome, resulting in substitutions, insertions, and/or deletions (indels) which can generate disruptive mutations. This system works well for disrupting a gene or eliminating a segment of DNA, both of which require DSBs in the target genome.

Unlike NHEJ, the HDR pathway can repair DSBs with high fidelity during the S and G2 phases of the cell cycle, enabling precise gene corrections using DNA donor templates. HDR employs a homologous single- or double-stranded DNA template to guide the repair process, which can be sourced from the host genome or provided externally for targeted genome editing. Research has shown that targeted in vivo gene integration can rectify mutations in genes such as *PDE6B*, *NR2E3*, *RPGR*, and *RPE65* [119,120,121]. However, HDR has limitations that make it less ideal for precise gene corrections in the retina. Cas9-induced DSBs often lead to significant indels, undermining the therapeutic potential of HDR. Additionally, the HDR pathway is mainly active in dividing cells, resulting in low efficiency in post-mitotic cells like photoreceptors and RPE cells. Consequently, HDR is generally unsuitable for precise genome editing in the eye.

Addressing IRDs caused by mutations in large genes exemplifies an advanced Cas9-based therapeutic approach in the retina. Mutations in the 7.4 kb *CEP290* gene are among the most frequent causes of LCA identified. The most common mutation in *CEP290* (c.2991+1655A>G) is a point mutation within an intron that creates an abnormal splice donor site, leading to the inclusion of 128 bp and the formation of a premature stop codon. Recent studies have shown that a single AAV-mediated delivery of Staphylococcus aureus Cas9 (SaCas9) with two sgRNAs can excise the aberrant splice donor created by the *CEP290* mutation in mouse and non-human primate models [122].

Several gene editing-based clinical trials for IRDs are currently in progress. One such trial involves using AAV vectors to deliver SaCas9 and CEP290-specific gRNAs to photoreceptor cells via subretinal injection. Editas Medicine sponsored a phase I/II clinical trial (NCT03872479) for LCA10, focusing on the safety, tolerability, and efficacy of their CRISPR/Cas9-based treatment, EDIT-101 [123]. This gene editing therapy employs AAV5 to deliver SaCas9 and CEP290-specific gRNAs, aiming to correct intron mutations in the *CEP290* gene [124].

The trial enrolled 14 patients with subretinal injections of EDIT-101 at varying doses: two adults at a low dose, five adults at an intermediate dose, five adults at a high dose, and two pediatric patients at the intermediate dose. Data from the phase I/II BRILLIANCE trial indicated that EDIT-101 was generally safe across all dose levels. Six patients experienced meaningful improvements from baseline in cone-mediated vision, while nine patients (64%) showed meaningful enhancements in BCVA, red light sensitivity, or mobility test scores [123]. However, only three patients exhibited clinically significant visual improvement, with two of these responders having homozygous mutations and just one of the twelve heterozygous patients responding positively. This indicates that EDIT-101 may be more effective in homozygous LCA10 patients compared to heterozygous ones.

Due to the limited number of eligible subjects, Editas Medicine has temporarily halted recruitment for the trial but will continue long-term follow-ups with the treated patients. Despite the challenges of a small patient pool and variable outcomes, EDIT-101 marks a notable advancement in gene editing for ocular diseases. Ongoing research will further refine gene editing methods to enhance their clinical applicability for these conditions.

Innovative Cas-based tools, including base editors (BEs) and prime editors (PEs), have emerged for precise genetic corrections in post-mitotic retinal cells without causing DSBs [125]. BEs utilize a modified catalytically impaired “nicking” Cas nuclease (dead Cas9) linked to DNA-modifying enzymes (cytidine or adenine deaminases) to facilitate the conversion of single bases, such as C to T or A to G [126]. These tools enable the accurate correction of point mutations or single-nucleotide polymorphisms at the target position of genomic DNA [127]. The use of split BE dual AAV vectors has shown therapeutic efficiency in achieving base editing in mouse retinal cells and a rodent model of RP [128,129,130]. Furthermore, single-AAV adenine base editor (ABE) systems have been created by reducing the size of ABEs and AAV components, thus enhancing in vivo targeting efficiency, editing effectiveness, lowering required AAV doses, and minimizing potential toxicity [131]. Given that many IRDs are caused by point mutations, base editors are expected to be widely applicable for therapeutic use.

Prime editors (PEs) signify a considerable advancement in CRISPR/Cas9-based gene editing. PEs also utilize a modified Cas9, specifically the Cas9-H840A nickase, to cut a single DNA strand and prevent DSB formation. The Cas9 nickase is linked to a reverse transcriptase, which enables the desired edit to be transcribed in reverse at the target site. PEs use a prime editing guide RNA (pegRNA) that contains both template sequences for reverse transcription and protospacer sequences. The Cas9-H840A nickase cleaves the target DNA strand, allowing the reverse transcriptase to synthesize a template DNA strand, thereby modifying the target site [132]. This method allows PEs to perform targeted genome modifications, including various substitutions and small insertions or deletions (indels), by leveraging cellular DNA repair mechanisms [132].

Several research groups have started using PEs for editing mutations associated with IRDs. In human cells, split PEs delivered via dual AAV1 vectors can mediate insertions and base conversions at multiple endogenous sites [133]. Two independent studies in rd12 mice have evaluated AAV-delivery of PE to edit the RPE65 mutation [134,135]. Subretinal injection of dual-AAVs carrying PE and pegRNA achieved a delivery efficiency of 23% and approximately 6.4% editing efficiency without any detectable indels, unintended substitutions, or off-target effects. Improved dark-adapted ERG responses, reaching up to 67% of the wild-type amplitude, were observed [134]. Another study using an optimized dual-AAV split-PE3 system administered via subretinal injection in rd12 mice showed 11.4 ± 2.3% editing in RPE cells, the restoration of RPE65 protein levels, and improved photoreceptor function and survival [135]. Although reports on the use of PEs in IRD gene therapy are still limited, they hold substantial promise for ocular gene therapy due to their extensive target site selection, high editing efficiency, and low off-target rates [135].

## 4. Challenges and Prospectives

As previously mentioned, gene-based therapy has shown great potential for addressing the genetic defects causing IRDs in children with INS. Currently, the majority of clinical trials for IRDs focus on AAV-based gene therapies, which target a variety of retinal conditions such as LCA, X-linked RP, achromatopsia, and X-linked retinoschisis. Innovations in AAV vector engineering, delivery methods, and safety improvements have significantly increased the effectiveness of these therapies, expanding their applicability to a wider range of retinal conditions.

Nevertheless, numerous challenges must be tackled before AAV-based gene therapy can become a truly curative treatment for IRDs [136]. Despite the limited packaging capacity of AAVs, key issues primarily pertain to safety, predictability, and the durability of the gene therapy outcomes. The genetic heterogeneity of IRDs requires the development of mutation-specific therapies, which is both time-consuming and costly. Ensuring long-term systemic efficacy and safety, particularly in terms of immune responses to AAV vectors, is crucial. Additionally, addressing manufacturing and regulatory challenges is essential for making these therapies widely available. Despite these hurdles, rapid progress in gene therapy techniques, coupled with a deeper understanding of the genetic and phenotypic diversity of IRDs, holds great promise for developing effective treatments.

The innovative CRISPR/Cas9 genome editing technology represents a promising approach for treating IRDs. Unlike AAV-mediated gene augmentation, CRISPR/Cas9 has the potential to address a broader spectrum of diseases with more enduring effects. However, efficient in vivo delivery remains a significant challenge for the clinical application of CRISPR/Cas therapeutics. This is because the system often requires two separate AAV vectors—one for the Cas endonuclease and another for the guide RNA (gRNA) expression cassette. Identifying smaller Cas9 orthologs that can be packaged together with the gRNA into a single AAV vector could help overcome this limitation. Additionally, the use of AAV vectors for long-term expression of CRISPR/Cas9 or base editors can result in significant off-target editing, which may reduce the precision of on-target gene edits. To address this, research efforts are increasingly focused on non-viral methods for the transient delivery of the editing machinery, such as nanoparticles [137] or engineered virus-like particles [138]. Furthermore, in vivo genome editing raises various safety and efficacy concerns. Therefore, the risks and benefits of genome editing therapies must be thoroughly evaluated to ensure their clinical viability.

In summary, combining precise genetic diagnostics with advanced AAV-based gene therapy presents a promising strategy for treating INS-associated IRDs. Future research should focus on broadening the range of treatable mutations, optimizing vector design, and guaranteeing the long-term safety and effectiveness of these therapies. By addressing these issues, we can advance toward effectively treating vision loss due to INS-associated IRDs, thus providing hope to affected families and contributing to the overarching goal of eliminating childhood blindness.

## 5. Conclusions

In conclusion, this review provides a comprehensive overview of the phenotypic and genetic landscape of IRDs, with a particular emphasis on recent advancements in understanding INS-associated IRDs. We have explored the potential of AAV-based gene therapy for retinal diseases, focusing on the clinical trials targeting INS-associated IRDs. A thorough understanding of the fundamental mechanisms of these diseases is essential for developing effective treatment. The diverse genotype–phenotype landscape of these conditions presents both obstacles and opportunities for therapeutic innovation.

High-throughput NGS technologies have significantly enhanced our understanding of the genetic underpinnings of INS-related IRDs, catalyzing the development of gene-based therapies for previously incurable conditions. The approval of Luxturna, the first AAV-based gene therapy, has paved the way for a significant number of gene-based therapies targeting INS-associated IRDs to enter late-stage clinical trials. These trials offer hope for improved vision and quality of life for pediatric patients, with additional approvals of gene-based therapies expected in the near future.

Despite ongoing challenges in AAV-based gene augmentation therapies, the continuous advancement of genetic therapeutic strategies—including gene editing therapies, capsid-engineered vectors, and delivery techniques—will be crucial in overcoming existing obstacles to effective and durable gene therapy for INS-associated IRDs. These advancements are poised to significantly transform the treatment paradigm for INS-associated IRDs.

## Figures and Tables

**Table 1:** Causative genes implicated in Leber congenital amaurosis (LCA).

Diseases	Gene	Protein	Function
LCA1	*GUCY2D*	Retinal guanylate cyclase-1	Phototransduction
LCA2	*RPE65*	Retinoid isomerohydrolase RPE65	Vision (retinoid) cycle
LCA3	*SPATA7*	Spermatogenesis associated protein 7	Photoreceptor ciliary transport
LCA4	*AIPL1*	Aryl-hydrocarbon interacting protein-like 1	Phototransduction
LCA5	*LCA5*	Lebercilin	Photoreceptor ciliary transport
LCA6	*RPGRIP1*	Retinitis pigmentosa GTPase regulator-interacting protein 1	Photoreceptor ciliary transport
LCA7	*CRX*	Cone–rod homeobox	Photoreceptor morphogenesis
LCA8	*CRB1*	Crumbs homologus 1	Photoreceptor morphogenesis
LCA9	*NMNAT1*	Nicotinamide nucleotide adenyltransferase 1	Coenzyme NAD biosynthesis
LCA10	*CEP290*	Centrosomal protein 290	Photoreceptor ciliary transport
LCA11	*IMPDH1*	Inosine 5′-monophosphate dehydrogenase 1	Guanine synthesis
LCA12	*RD3*	Retinal Degeneration 3	Photoreceptor ciliary transport
LCA13	*RDH12*	Retinol dehydrogenase 12	Visual (retinoid) cycle
LCA14	*LRAT*	Lecithin:retinol acyltransferase	Visual (retinoid) cycle
LCA15	*TULP1*	Tubby-like protein	Photoreceptor ciliary transport
LCA16	*KCNJ13*	Potassium inwardly rectifying channel subfamily J member 13	Signal transduction
LCA17	*CABP4*	Calcium-binding protein 4	Signal transduction
	*GDF6*	Growth differentiation factor 6	Photoreceptor morphogenesis
LCA18	*PRPH2*	Peripherin 2	Photoreceptor morphogenesis
LCA19	*USP45*	ubiquitin specific peptidase 45	deubiquitylation
Unclassified	*CCT2*	Chaperonin Containing TCP1 Subunit 2	Photoreceptor ciliary transport
	*CLUAP1*	Clusterin associated protein 1	Photoreceptor morphogenesis
	*DTHD1*	Death-domain containing protein 1	unknown
	*IQCB1*	IQ motif containing B1 protein	Photoreceptor ciliary transport
	*OTX2*	Orthodenticle homeobox 2 protein	Photoreceptor morphogenesis

**Table 2:** Types and targeted gene inheritance patterns of congenital stationary night blindness (CSNB).

Inheritance Pattern	Types	Genes	Gene ID (OMIM^#^)
Autosomal Dominant	Riggs	*RHO*	180380
		*GNAT1*	610444
		*PDE6B*	180072
	Abnormal fundus	*SAG*	181031
		*GRK1*	613411
		*RDH5*	610617
		*RLBP1*	180090
		*RPE65*	180069
Autosomal Recessive	Complete	*GRM6*	604096
		*TRPM1*	603576
		*RIMS2*	606630
		*GPR179*	414515
		*GNB3*	617024
		*LRIT3*	615004
	Incomplete	*CABP4*	608965
		*CACNA2D4*	608171
	Riggs	*SLC24A1*	613830
		*GNAT1*	610444
X-linked	Complete	*NYX*	300278
	Incomplete	*CACNA1F*	300110

**Table 3:** Clinical trials of targeted gene therapy for INS-associated IRDs.

Gene	Mutation	Treatment (Sponsor)	Agent (Constructs)	Function	Delivery Route	Clinical Trial ID	Trial Phase
Leber Congenital Amaurosis (LCA)							
*GUCY2D*	Loss of Function	Atsena Therapeutics (ATSN-101)	AAV8-GRK1-GUCY2D	Restores function	Subretinal	NCT03920007	I/II
*CEP290*	c.2991+ 1655A>G	Editas (EDIT-101)	AAV5-GRK1-Cas9	Gene editing	Subretinal	NCT03872479 BRILLANCE	I/II
*CEP290*	c.2991+ 1655A>G	ProQR Therapeutics	QR-110 (Sepofarsen)	Normal mRNA (AON)	Intravitreal	NCT03913143NCT04855045 NCT03140969	II/III II/III I/II
*LCA5*	Loss of Function	Opus Genetics	AAV8.hLCA5 (OPGx-001)	Restores function	Subretinal	NCT05616793	I/II
*RPE65*	Loss of Function	Janssen/MeiraGTx UK II Ltd.	AAV5-PRE65	Restores function	Subretinal	NTC02781480	I/II
			AAV6-RPE65			NCT02946879	I/II
*RPE65*	Loss of Function	HuidaGene Therapeutics	rAAV2-RPE65 rAAV9-RPE65	Restores function	Subretinal	NCT05906953 NCT06088992	I/II I
*RPE65*	Loss of Function	Spark Therapeutics	AAV2-hRPE65v2	Restores functional protein	Subretinal	NCT00999609	III
			AAV2-hRPE65v2			NCT01208389	I/II
			AAV2-hRPE65v2			NCT03602820	III
*RPE65*	Loss of Function	AGTC	rAAV2-CB-hRPE65	Restores function	Subretinal	NCT00749957	I/II
*RPE65*	Loss of Function	Nantes University Hospital	rAAV2/4.hRPE65	Restores function	Subretinal	NCT01496040	I/II
*RPE65*	Loss of Function	University of Pennsylvania	rAAV-CBSB-hRPE65	Restores function	Subretinal	NCT00481546	I/II
Achromatopsia (ACHM)							
*GNCA3*	Loss of Function	Janssen/ STZ eyetrial	rAAV2/8.hCNGA3	Restores function	Subretinal	NCT02610582	I/II
*GNCA3*	Loss of Function	AGTC (Beacon Therapeutics)	rAAV2tYF- PR1.7-hCNGA3	Restores function	Subretinal	NCT02935517	I/II
*GNCA3*	Loss of Function	Janssen/MeiraGTx UK II Ltd.	AAV2/8-hG1.7p. coCNGA3	Restores function	Subretinal	NCT03758404	I/II
*GNCB3*	Loss of Function	Janssen/MeiraGTx UK II Ltd.	rAAV2/8.hCNGB3	Restores function	Subretinal	NCT03758404	I/II
*GNCB3*	Loss of Function	AGTC (Beacon Therapeutics)	rAAV2tYF- PR1.7-hCNGB3	Restores function	Subretinal	NCT02599922	I/II
*GNCB3*	Loss of Function	Janssen/MeiraGTx UK II Ltd.	AAV2/8-hG1.7p. coCNGB3	Restores function	Subretinal	NCT03001310	I/II
X-linked Retinoschisis (XL-RS)							
*RS1*	Loss of Function	AGTC	rAAV2YF-CB-hRS1	Restores function	Intravitreal	NCT02416622	I/II
*RS1*	Loss of Function	Atsena Therapeutics	ATSN-201 (AAV.SPR.hRS1)	Restores function	Intravitreal	NCT05878860	I/II
*RS1*	Loss of Function	InnoVec Biotherapeutics	IVB102	Restores function	Intravitreal	NCT06289452	I/II
*RS1*	Loss of Function	Shanghai General Hospital	LX103	Restores function	Intravitreal	NCT05814952	I/II
*RS1*	Loss of Function	West China Hospital	JWK002	Restores function	Intravitreal	NCT06345898	I/II
*RS1*	Loss of Function	National Eye Institute	AAV8-scRS/ IRBPhRS	Restores function	Intravitreal	NCT02317887	I/II
X-linked Retinitis Pigmentosa (XL-RP)							
*RPGR*	Loss of Function	Janssen	AAV5-hRKp.RPGR	Restores function	Subretinal	NCT04671443 (LUMEOS)	III
*RPGR*	Loss of Function	AGTC (Beacon Therapeutics)	rAAV2tYF-GRK1-RPGRco	Restores function	Subretinal	NCT06333249 (SKYLINE)	II
*RPGR*	Loss of Function	AGTC (Beacon Therapeutics)	rAAV2tYF-GRK1-RPGRco	Restores function	Subretinal	NCT04850118 (VISTA)	II/III
*RPGR*	Loss of Function	4D Molecular Therapeutics	AAV.R100-hcoRPGR (4D-125)	Restores function	Intravitreal	NCT04517149 (EXCEL)	I/II
*RPGR*	Loss of Function	Janssen	AAV5-hRKp.RPGR	Restores function	Subretinal	NCT05926583 NCT04794101	III III
*RPGR*	Loss of Function	Frontera Therapeutics	FT-002	Restores function	Intravitreal	NCT06492850	I/II
*RPGR*	Loss of Function	Biogen	AAV8-RPGR (BIIB112)	Restores function	Subretinal	NCT03584165 (SOLSTICE)	III
Autosomal dominant Retinitis Pigmentosa (adRP)							
*RHO*	P23H	ProQR Therapeutics	QR-1123	Gene silencing	Intravitreal	NCT04123626 (AURORA)	I/II
*RHO*	Optogenetic	AbbVie	AAV2-ChR2 (RST-001)	Gene agnostic	Intravitreal	NCT02556736	I/II
*RHO*	Agnostic	SparingVision	AAV-RdCVF-RdCVFL (SPVN06)	Gene agnostic	Subretinal	NCT05748873	I/II
*RHO*	Genetic modifier	Ocugen	AAV5-NR2E3 (OCU400-301)	Gene agnostic	Subretinal	NCT06388200	III
Autosomal recessive Retinitis Pigmentosa (arRP)							
*RLBP1*	Loss of Function	Novartis Therapeutics	AAV8-RLBP1 (CPK850)	Restores function	Subretinal	NCT03374657	I/II
*USH2A*	USH2A (*exon 3*)	Laboratoires Thea	Ultevursen (QR-421a)	Gene silencing	Intravitreal	NCT05176717 NCT05158296	II/III
*PDE6A*	Loss of Function	STZ eyetrial	rAAV-hPDE6A	Restores function	Subretinal	NCT04611503	I/II
*PDE6B*	Loss of Function	Coave Therapeutics	AAV5-hPDE6B	Restore function	Subretinal	NCT03328130	I/II
*CNGA1*	Loss of Function	ViGeneron GmbH	AAV2.NN-CNGA1 (VG-901)	Restore function	Intravitreal	NCT06291935	I
*MERTK*	Loss of Function	Fowzan Alkuraya	AAV2-MERTK	Restores function	Subretinal	NCT01482195	I

## Data Availability

Not applicable.

## Supplementary Materials

The following supporting information can be downloaded at: https://www.mdpi.com/article/10.3390/life14111356/s1: Table S1: INS-associated IRDs and their corresponding causative genes. Table S2: INS-associated IRD genes and their corresponding inheritance patterns and phenotypes.
