# Supplementary material for: Infantile Nystagmus Syndrome—Associated Inherited Retinal Diseases: Perspectives from Gene Therapy Clinical Trials
Source: Life (Basel). 2024 Oct 23;14(11):1356. doi: 10.3390/life14111356 (PMC11595273; doi:10.3390/life14111356)
Supplement: Supplementary file 1 [file life-14-01356-s001.zip › life-3105550-supplementary.pdf]

Supplementary Table 1: INS-associated IRDs and their corresponding causative genes

Disease	# of Genes	Gene Names
AR-ACHM	6	*ATF7, CNGA3, CNGB3, GNAT2, PDE6C, PDE6H*
AR-BBS	25	*ADIPOR1, ARL6, BBIP1, BBS1, BBS2, BBS4, BBS5, BBS7, BBS9, BBS10, BBS12, C8orf37, CEP19, CEP290, IFT172, IFT27, INPP5E, KCNJ13, LZTFL1, MKKS, MKS1, NPHP1, SDCCAG8, TRIM32, TTC8*
AD-CD/CRD	6	*CRX, GUCA1A, GUCY2D, PROM1, PRPH2, RAX2*
AR-CD/CRD	16	*ABCA4, ADAM9, ALMS1, C21orf2,CACNA2D4, CDHR1, CERKL, CNGA3, CNGB3, IFT81, KCNV2, POC1B, RAB28, RAX2, RPGRIP1, TTLL5*
XL-CD/CRD	2	*CACNA1F, RPGR*
AD-CSNB	3	*GNAT1, PDE6B, RHO*
AR-CSNB	11	*CABP4, GNAT1, GNB3, GPR179, GRK1, GRM6, LRIT3, RDH5, SAG, SLC24A1, TRPM1*
XL-CSNB	2	*CACNA1F, NYX*
AD-LCA	3	*CRX, IMPDH1, OTX2*
AR-LCA	23	*AIPL1, CABP4, CCT2, CEP290, CLUAP1, CRB1, CRX, GDF6, GUCY2D, IFT140, INPP5E,IQCB1, KCNJ13, LCA5, LRAT, NMNAT1, PRPH2, RD3, RDH12, RPE65, RPGRIP1, SPATA7, TULP1*
AD-Optic atrophy	3	*AFG3L2, MFN2, OPA1*
AR-Optic atrophy	4	*ACO2, NBAS, RTN4IP1, TMEM126A*
AD-OA-syndromic	2	*ELOVL1, MFN2, NR2F1*
AR-OA-syndromic	3	*ACO2, C12orf65, OPA3*
XL-OA	1	*TIMM8A*
AD-Other IRDs	13	*BEST1, CAPN5, FZD4, ITM2B, LRP5, MAPKAPK3, MIR204, OPN1SW, PAX6, RCBTB1, TEAD1, TSPAN12, ZNF408*
AR-Other IRDs	19	*BEST1, CYP4V2, LRP5, MFRP, NBAS, NR2E3, NRL, OAT, P3H2, PLA2G5, PROM1, RBP4, RCBTB1, RGS9, RGS9BP, RLBP1, SLC38A8, TSPAN12, VSX2*
XL-Other IRDs	5	*CHM, NDP, OPN1LW, OPN1MW, RS1*
AD-RP	26	*ADIPOR1, ARL3, BEST1, CA4, CRX, HK1, IMPDH1, IMPG1, KLHL7, NR2E3, NRL, PRPF3, PRPF4, PRPF6, PRPF8, PRPF31, PRPH2, RDH12, RHO, RP1, RP9, RPE65, SAG, SNRNP200, SPP2, TOPORS*
AR-RP	68	*ABCA4, AGBL5, AHR, ARHGEF18, ARL6, ARL2BP, ASRGL1,BBS1, BBS2, BEST1, C2orf71, C8orf37, C21orf2, CERKL, CLCC1, CNGA1, CNGB1, CRB1, CYP4V2, DHDDS, DHX38, EMC1, EYS, FAM161A, GPR125, HGSNAT, IDH3A, IDH3B, IFT140, IFT172, IMPG2, KIAA1549, KIZ, LRAT, MAK, MERTK, MVK, NEK2, NEUROD1, NR2E3, NRL, PDE6A, PDE6B, PDE6G, POMGNT1, PRCD, PROM1, RBP3, REEP6, RGR, RHO, RLBP1, RP1, RP1L1, RPE65, SAG, SAMD11, SCAPER,SLC4A7,SLC7A14, SNRNP200, SPATA7, TRNT1, TTC8, TULP1, USH2A, ZNF408, ZNF513*
XL-RP	3	*OFD1, RP2, RPGR*

Supplementary Table 2: INS-associated IRD genes and their corresponding inheritance patterns and phenotypes

Genes HGNC	Transcript name	Inheritance	Disease/phenotype	Disease ID (OMIM#)	Gene ID (OMIM#)
ABCA4	NM_000350	AR	STGD, CRD, maculopathy, RP	248200	601691
ACBD5	NM_145698	AR	CRD- syndromic	618863	616618
ACO2	NM_001098	AR	OA, OA- syndromic	614559	100850
ADAM9	NM_003816	AR	CRD	612775	602713
ADAMTS18	NM_199355	AR	Knobloch, Microcornea	615458	607512
ADGRV1	NM_032119	AR	USH2C	605472	602851
AFG3L2	NM_006796	AD/AR	OA, OA- syndromic	618977	604581
AGBL5	NM_001035507	AR	RP	617023	615900
AHI1	NM_001134830	AR	Joubert syndrome, RP	608629	608894
AIPL1	NM_014336	AR	LCA,CRD	604393	604392
ALMS1	NM_015120	AR	Alström Syndrome, CRD	203800	606844
ARHGEF18	NM_015318	AR	RP	617433	616432
ARL2BP	NM_012106	AR	RP	615434	615407
ARL3	NM_004311	AD	RP	618161	604695
ARL6	NM_001278293	AR	BBS, RP	600151	608845
ARSG	NM_014960	AR	USH	618144	610008
ATF6	NM_007348	AR	ACHM	616517	605537
BBIP1	NM_001243783	AR	BBS	615995	613605
BBS1	NM_024649	AR	BBS, RP	209900	209901
BBS10	NM_024685	AR	BBS	615987	610148
BBS12	NM_152618	AR	BBS	615989	610683
BBS2	NM_031885	AR	BBS, RP	615981	606151
BBS4	NM_033028	AR	BBS	615982	600374
BBS5	NM_152384	AR	BBS	615983	603650
BBS7	NM_176824	AR	BBS	615984	607590
BBS9	NM_001033604	AR	BBS	615986	607968
BEST1	NM_001139443	AR/AD	Best, RP, other RD	611809	607854
C12orf65	NM_152269	AR	OA- syndromic	613559	613541
C5orf42	NM_023073	AR	Joubert Syndrome	614615	614571
C8orf37	NM_177965	AR	BBS, RP	617406	614477
CABP4	NM_145200	AR	CSNB, LCA, CRD	610427	608965

CACNA1F	NM_001256789	XL	CSNB,CRD	300071	300110
CACNA2D4	NM_172364	AR	CRD	610478	608171
CC2D2A	NM_001080522	AR	Joubert Syndrome	216360	612013
CDH23	NM_022124	AR	USH	601067	605516
CDHR1	NM_033100	AR	CRD	613660	609502
CEP164	NM_001271933	AR	Senior Löken Syndrome	614845	614848
CEP250	NM_007186	AR	USH	618358	609689
CEP290	NM_025114	AR	LCA, BBS, RD-syndromic	611755	610142
CEP78	NM_032171	AR	CRD	617236	617110
CERKL	NM_001030311	AR	CRD,RP	608380	608381
CFH	NM_000186	AD	Maculopathy	610698	134370
CLCC1	NM_001048210	AR	RP	609913	617539
CLRN1	NM_001195794	AR	USH	276902	606397
CNGA1	NM_001142564	AR	RP	613756	123825
CNGA3	NM_001298	AR	ACHM, CRD	216900	600053
CNGB1	NM_001297	AR	RP	613767	600724
CNGB3	NM_019098	AR	ACHM, CRD	262300	605080
CNNM4	NM_020184	AR	Jalili Syndrome	217080	607805
COL11A1	NM_001854	AD	Stickler Syndrome	604841	120280
COL18A1	NM_030582	AR	Knobloch Syndrome	267750	120328
COL2A1	NM_001844	AR/AD	Stickler Syndrome	108300	120140
COL9A1	NM_001851	AR	Stickler Syndrome	614134	120210
CRB1	NM_001257965	AR	LCA, RP	613835	604210
CRX	NM_000554	AR/AD	CRD, LCA, RP	120970	602225
CSPP1	NM_024790	AR	Joubert Syndrome	615636	611654
CTNNA1	NM_001903	AD	Maculopathy	608970	116805
CWC27	NM_005869	AR	RP- syndromic	250410	617170
CYP4V2	NM_207352	AR	Bietti, RP	210370	608614
DFNB31	NM_001083885	AR	USH	611383	607928
DHX38	NM_014003	AR	RP	618220	605584
DRAM2	NM_178454	AR	Maculopathy	616502	613360
EFEMP1	NM_001039349	AD	Maculopathy	126600	601548
ELOVL1	NM_001256402	AD	OA- syndromic	618527	611813
ELOVL4	NM_022726	AD	Maculopathy	600110	605512
EMC1	NM_015047	AR	RP	616875	616846
ESPN	NM_031475	AR	USH	609006	606351
EXOSC2	NM_014285	AR	RP- syndromic	617763	602238

EYS	NM_001142800	AR	RP	602772	612424
FAM161A	NM_001201543	AR	RP	606068	613596
FLVCR1	NM_014053	AR	RP- syndromic	609033	609144
GDF6	NM_001001557	AR	LCA	615360	601147
GNAT1	NM_144499	AR/AD	CSNB	616389	139330
GNAT2	NM_005272	AR	ACHM	613856	139340
GNB3	NM_002075	AR	CSNB	617024	139130
GPR179	NM_001004334	AR	CSNB	614565	414515
GRK1	NM_002929	AR	CSNB	613411	180381
GRM6	NM_000843	AR	CSNB	257270	604096
GUCA1A	NM_000409	AD	CRD	602093	600364
GUCY2D	NM_000180	AR/AD	CRD, LCA	204000	600179
HARS	NM_001258042	AR	USH	614504	142810
HGSNAT	NM_152419	AR	RP, RP-syndromic	616544	610453
HK1	NM_033500	AD	RP	617460	142600
HMCN1	NM_031935	AD	Maculopathy	603075	608548
IDH3A	NM_005530	AR	RP	619007	601149
IDH3B	NM_006899	AR	RP	612572	604526
IFT140	NM_014714	AR	LCA, RP, RD-syndromic	266920	614620
IFT172	NM_015662	AR	RP, BBS	616394	607386
IFT27	NM_006860	AR	BBS	615996	615870
IMPDH1	NM_183243	AD	RP,LCA	613837	146690
IMPG1	NM_001563	AR/AD	Maculopathy, RP	616151	602870
IMPG2	NM_016247	AR	RP	613581	607056
INPP5E	NM_019892	AR	BBS, RD-syndromic, LCA	610156	613037
IQCB1	NM_001023570	AR	LCA	609254	609237
ITM2B	NM_021999	AD	RD- other	616079	603904
JAG1	NM_000214	AD	Alagille syndrome	118450	601920
KCNJ13	NM_002242	AR/AD	BBS, LCA, RD-syndromic	614186	603208
KIZ	NM_018474	AR	RP	615780	615757
KLHL7	NM_001172428	AD	RP	612943	611119
LCA5	NM_001122769	AR	LCA	604537	611408
LRAT	NM_004744	AR	LCA, RP	613341	604863
LRIT3	NM_198506	AR	CSNB	615058	615004
LZTFL1	NM_001276379	AR	BBS	615994	606568
MAK	NM_001242957	AR	RP	614181	154235
MERTK	NM_006343	AR	RP	613862	604705

MFN2	NM_014874	AD	OA, OA- syndromic	609260	608507
MFRP	NM_031433	AR	RD-other	611040	606227
MFSD8	NM_001371596	AR	Maculopathy	616170	61124
MIR204	NR_029621	AD	Retinal dystrophy with iris coloboma	616722	610942
MKKS	NM_170784	AR	BBS	605231	604896
MKS1	NM_017777	AR	BBS	615990	609883
MYO7A	NM_001127179	AR	USH1	276900	276903
NBAS	NM_015909	AR	OA, RD-other	614800	608025
NEK2	NM_001204182	AR	RP	615565	604043
NMNAT1	NM_022787	AR	LCA	608553	608700
NPHP1	NM_000272	AR	Joubert	609583	607100
NR2E3	NM_016346	AR/AD	RP, ESCS, CPRD	611131	604485
NR2F1	NM_005654	AD	OA-syndromic	615722	132890
NRL	NM_006177	AR/AD	RP, ESCS	613750	162080
NYX	NM_022567	XL	CSNB	310500	300278
OFD1	NM_001330209	XL	RP, Joubert Syndrome	300424	300170
OPA1	NM_130837	AD	OA	165500	605290
OPA3	NM_001017989	AR	OA-syndromic	165300	606580
OPN1LW	NM_020061	XL	BCM	303700	300822
OPN1MW	NM_000513	XL	BCM	303700	300821
OTX2	NM_021728	AD	LCA, pattern dystrophy	610125	600037
P3H2	NM_018192	AR	High myopia, retinal degeneration	614292	610341
PAX2	NM_003987	AD	renal-coloboma syndrome	120330	167409
PAX6	NM_000280	AD	Aniridia, foveal hypoplasia	136520	607108
PCDH15	NM_033056	AR	USH1	601067	605514
PDE6A	NM_000440	AR	RP	613810	180071
PDE6B	NM_000283	AR/AD	CSNB, RP	613801	180072
PDE6C	NM_006204	AR	ACHM	613093	600827
PDE6G	NM_002602	AR	RP	613582	180073
PDE6H	NM_006205	AR	ACHM	610024	601190
PLA2G5	NM_000929	AR	LORD	228980	601192
PLK4	NM_014264	AR	RD- syndromic	616171	605031
POC1B	NM_172240	AR	CRD,RD-syndromic	615973	614784
PROM1	NM_001145849	AR/AD	CRD, maculopathy, RP	612657	604365

PRPF3	NM_004698	AD	RP	601414	607301
PRPF31	NM_015629	AD	RP	600138	606419
PRPF4	NM_004697	AD	RP	615922	607795
PRPH2	NM_000322	AR/AD	RP, CRD, LCA	608133	179605
PRPS1	NM_002764	XL	RD- syndromic	311070	311850
RAB28	NM_001017979	AR	CRD	615374	612994
RAX2	NM_032753	AD	CRD	610381	610362
RBP3	NM_002900	AR	RP	268000	180290
RCBTB1	NM_018191	AR	RD- other	617175	607867
RD3	NM_001033560	AR	LCA	610612	180040
RDH11	NM_001252650	AR	RD- syndromic	616108	607849
RDH12	NM_152443	AR/AD	LCA, RP	612712	608830
RDH5	NM_001199771	AR	FA, CD	136880	601617
RHO	NM_000539	AR/AD	RP, CSNB	613731	180380
RIMS2	NM_001348484	AR	Syndromic CSNB	618970	606630
RLBP1	NM_000326	AR	RP, RPA	607475	180090
ROM1	NM_000327	DIGENIC	RP	608133	180721
RP1	NM_006269	AR/AD	RP	180100	603937
RP1L1	NM_178857	AR/AD	Maculopathy, RP	613587	608581
RP2	NM_006915	XL	RP	312600	300757
RP9	NM_203288	AD	RP	180104	607331
RPE65	NM_000329	AR/AD	LCA, RP	204100	180069
RPGR	NM_000328	XL	CRD, XL- RP,	304020	312610
RPGRIP1	NM_020366	AR	CRD, LCA, RD- syndromic	613826	605446
RPGRIP1L	NM_001127897	AR	Joubert Syndrome	611560	610937
RS1	NM_000330	XL	Retinoschisis	312700	300839
RTN4IP1	NM_032730	AR	OA	616732	610502
SAG	NM_000541	AR/AD	RP,CSNB	613758	181031
SCAPER	NM_020843	AR	RP	618195	611611
SDCCAG8	NM_006642	AR	BBS, RP-syndromic	615993	613524
SLC24A1	NM_004727	AR	CSNB	613830	603617
SLC25A46	NM_138773	AR	RD- syndromic	619303	610826
SLC38A8	NM_001080442	AR	Foveal hypoplasia	609218	615585
SLC7A14	NM_020949	AR	RP	615725	615720
SNRNP200	NM_014014	AD/AR	RP	610359	601664
SPATA7	NM_018418	AR	LCA,RP	604232	609868
TIMP3	NM_000362	AD	Maculopathy	136900	188826
TMEM126A	NM_032273	AR	OA	612989	612988

TMEM216	NM_001173990	AR	Joubert Syndrome	608091	613277
TMEM231	NM_001077416	AR	RP-syndromic	614970	614949
TMEM237	NM_001044385	AR	Joubert Syndrome	614424	614423
TOPORS	NM_005802	AD	RP	609923	609507
TRIM32	NM_012210	AR	BBS	615988	602290
TRNT1	NM_182916	AR	RP,RP-syndromic	616959	612907
TRPM1	NM_001252020	AR	CSNB	613216	603576
TTC8	NM_144596	AR	BBS,RP,CRD	615985	608132
TTLL5	NM_015072	AR	CRD	615860	612268
TUB	NM_177972	AR	RD- syndromic	616188	601197
TULP1	NM_003322	AR	LCA,RP	613843	602280
USH1C	NM_005709	AR	USH1, atypical USH	276904	605242
USH1G	NM_173477	AR	USH1	606943	607696
USH2A	NM_206933	AR	USH2, RP	276901	608400
VCAN	NM_004385	AD	Wagner disease	143200	118661
VSX2	NM_182894	AR	Microphthalmia	610093	142993
WFS1	NM_006005	AR	Wolfram Syndrome	222300	606201
ZNF423	NM_001271620	AR	Joubert Syndrome	614844	604557
